# Biological function and clinical application prospect of tsRNAs in digestive system biology and pathology

**DOI:** 10.1186/s12964-023-01341-8

**Published:** 2023-10-30

**Authors:** Juan Du, Tianyi Huang, Zhen Zheng, Shuai Fang, Hongxia Deng, Kaitai Liu

**Affiliations:** 1 Health Science Center, Ningbo University, Ningbo, 315211 Zhejiang China; 2 Department of Radiation Oncology, The Affiliated Lihuili Hospital of Ningbo University, Ningbo, Zhejiang China; 3 The Affiliated Hospital of Medical School of Ningbo University, Ningbo, Zhejiang China; 4 The Affiliated Lihuili Hospital of Ningbo University, Ningbo, Zhejiang China

**Keywords:** tsRNAs, Digestive disease, Diagnosis, Prognosis, Biomarkers

## Abstract

tsRNAs are small non-coding RNAs originating from tRNA that play important roles in a variety of physiological activities such as RNA silencing, ribosome biogenesis, retrotransposition, and epigenetic inheritance, as well as involvement in cellular differentiation, proliferation, and apoptosis. tsRNA-related abnormalities have a significant influence on the onset, development, and progression of numerous human diseases, including malignant tumors through affecting the cell cycle and specific signaling molecules. This review introduced origins together with tsRNAs classification, providing a summary for regulatory mechanism and physiological function while dysfunctional effect of tsRNAs in digestive system diseases, focusing on the clinical prospects of tsRNAs for diagnostic and prognostic biomarkers.

Video Abstract

**Supplementary Information:**

The online version contains supplementary material available at 10.1186/s12964-023-01341-8.

## Introduction

tRNA-derived small noncoding RNAs (tsRNAs), members of the noncoding RNA (ncRNA) family, can be categorized into stress-driven RNAs (tiRNAs) and tRNA fragments (tRFs) [1]. tRFs can be further separated into 5'-tRF and 3'-tRF depending upon the the presence of 5 'or 3' sequences, while tiRNA can be segregated within 5'-tiRNA and 3'-tiRNA [2]. According to the restriction site, tRFs can be cleaved into many subtypes, such as tRF-5a, tRF-5b, tRF-5c, tRF-3a, tRF-3b, and tRF-2 [3]. tsRNAs play different biological functions under different regulatory mechanisms and affect various stages of human disease progression [4]. At the same time, under the regulation of various signaling molecules, tsRNAs are involved in genetic and epigenetic regulation, RNA silencing, transcriptional and reverse transcriptional regulation, RNA stability regulation, and translation regulation [5–8]. Early research has established the presence of tsRNAs in bacteria, eukaryotes, and archaea [9, 10]. Researchers have discovered that tsRNAs are commonly prevalent in various species, including yeast, *Trypanosoma brucei*, Arabidopsis, and mice, due to the quick advances in sequencing technology [11–14].

Recent research has revealed that the biological function of tsRNAs is closely related to multiple biological processes, particularly in human cells. Numerous illnesses (cancer and noncancer diseases) in humans develop and progress as a result of the dysregulation of tsRNAs, such as tRF-23-Q99P9P9NDD in gastric cancer, tRF-26-P4R8YP9LOND in colorectal cancer, tRF-Gly-TCC-016 in oral fibrosis, tRF3-Thr-AGT in acute pancreatitis [15–18]. Digestive system illnesses are among the most frequent within the clinical setting, and their prevalence has gradually increased [19]. At least 100,000 patients are treated for gastrointestinal disorders for the first time each year [20]. In particular, digestive system cancers are characterized by high morbidity and mortality [21]. The digestive system is the site for four of the top ten cancers worldwide, namely, hepatocellular carcinoma (HCC), gastric cancer (GC), esophageal cancer (EC), colorectal cancer (CRC). Among the top ten cause of cancer-related deaths, half are digestive system tumors: CRC, HCC, GC, EC, and pancreatic cancer (PC) [22].

Therefore, digestive system cancers are a main contributor cancer-related fatalities globally [23–27]. With the detection of tumor markers and the popularization of gastroscopy and colonoscopy medical technology, as well as the application of surgical treatment, chemoradiotherapy, targeted therapy, immunotherapy, and comprehensive treatment strategies, the prognosis of digestive system cancer has relatively improved, but long-termtherapeutic effects are still not satisfactory, which seriously threatens human health [28, 29]. Therefore, increasing the overall survival rate of intestinal cancers requires early detection during the development of new anticancer drugs. Recently, several investigations validated that tsRNAs have a crucial biological function within various digestive system cancers, including EC, GC, CRC, PC, oral cancer, hypopharyngeal cancer, and hepatobiliary system tumors [30–36]. tsRNAs are commonly located within plasma/serum. This presents a significant advantage for noninvasive cancer diagnosis, so tsRNAs are anticipated to serve as a biomarker for detecting cancer at an early stage [15, 34]. Moreover, thisinvestigation demonstrates that tsRNAs have important application prospects in evaluating cancer prognosis and as novel therapeutic targets [37]. Furthermore, tsRNAs make a significant biological contribution to benign digestive system diseases, such as oral fibrosis, oral flora disturbance, liver injury, fatty liver, and pancreatitis, and have good clinical application prospects [17, 18, 38–40].

This review article discusses the origin, classification, regulatory mechanism and biological function of tsRNAs in digestive system diseases. We concentrated on the clinical potential of tsRNAs as a diagnostic and prognostic molecular markers in digestive system diseases.

## Origin and classification of tsRNAs

**Fig. 1:**
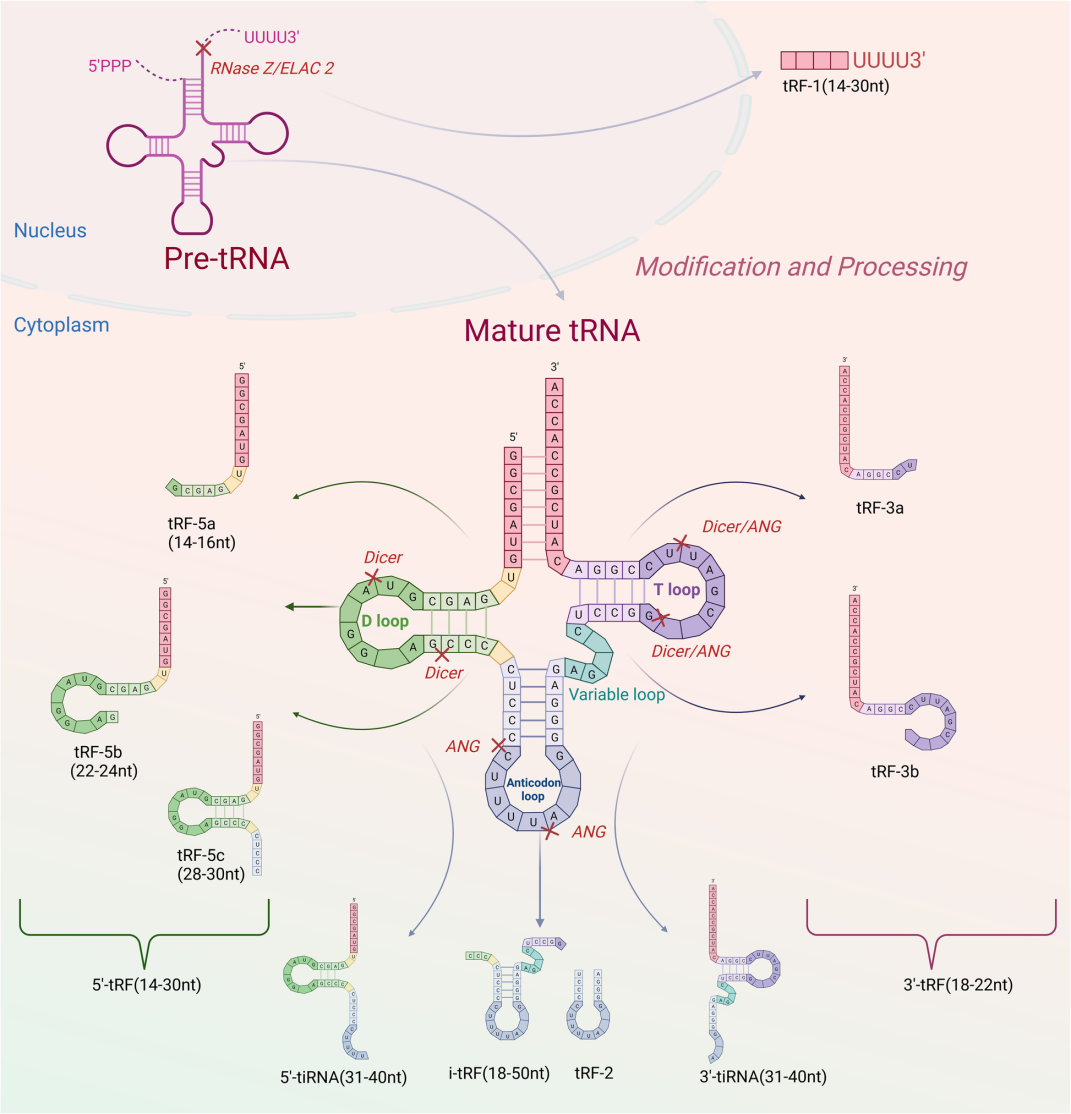
Origin and classification of tsRNAs. tRFs that are derived from the 5' and 3' ends are called 5'-tRF and 3'-tRF. In the pre-tRNAs in the nucleus, RNase Z-specific endonuclear cleavage catalyzes the 3 'end to produce tRF-1. In the mature structure of tRNA, 5'-tRF can be cleaved at the cleavage site on the D loop, which includes tRF-5a, tRF-5b and tRF-5c. 3'-tRF can be generated at the cleavage site on the T loop, including tRF-3a and tRF-3b, 5'-tRFsand 3'-3'-tRF. tiRNAwere form ed by cutting at sites in the anticodon loop, which could be divided into 5'-tiRNA and 3'-tiRNA. i-tRF and tRF-2 were formed by cutting at two sites simultaneously in the T loop and the D loop

tsRNAs, derived from tRNAs, are short-chain ncRNAs of 18–40 nucleotides (nt) in length that areformed through the processing of mature tRNAs or precursor of tRNAs (pre-tRNAs) [7]. Different tRNA sources and enzyme-cutting sites allow tRNAs to form a range of tsRNAs with various structures and roles that fall under tRFs and tiRNAs [41]. Among these tRFs, 5'-tRF (14–30 nt) and 3'-tRF (18–22 nt) are randomly generated through the 5' and 3' ends, respectively [42–44]. As shown in Fig. 1, during the maturation of tRNA, the 5' and 3' ends of pre-tRNAs within the nucleus are excised under the influence of RNase P together with RNase Z; accordingly [45, 46], RNase Z-specific endonuclease cleavage catalyzes the 3' end to produce tRF-1 (14–30 nt) [47]. The cleavage sites on the D loop, which includes tRF-5a (14–16 nt), tRF-5b (22–24 nt), and tRF-5c (28–30 nt), together with the T loop, which includes tRF-3a and tRF-3b, 5'-tRF, and 3'-tRF, reflect where the mature structure of tRNA produces 5'-tRF [48]. tiRNAs, which are 31–40 nucleotides long, are produced by cleaving the anticodon loop. These tiRNAs are typically classified as 5'-tiRNA and 3'-tiRNA halves. i-tRF (18–50 nt) and tRF-2 are generated through simultaneous cleavage at two sites within the T and D loops [49].

## tsRNAs in digestive system diseases

Numerous unique tsRNAs have been discovered recently using sequencing and genome technologies thanks to bioinformatics' popularization and advanced technology [50]. They can be found in large quantities in bodily fluids such as semen, urine, serum, and plasma [34, 51–53]. Several investigations have provided evidence that the dysregulated tsRNA expression profiles are linked to the initiation/progress of digestive system disorders [54]. We discuss the progression of tsRNA-related research from two perspectives in this section: cancers and mild conditions for digestive system.

### tsRNAs in digestive system cancers

#### tsRNAs in GC

**Fig. 2:**
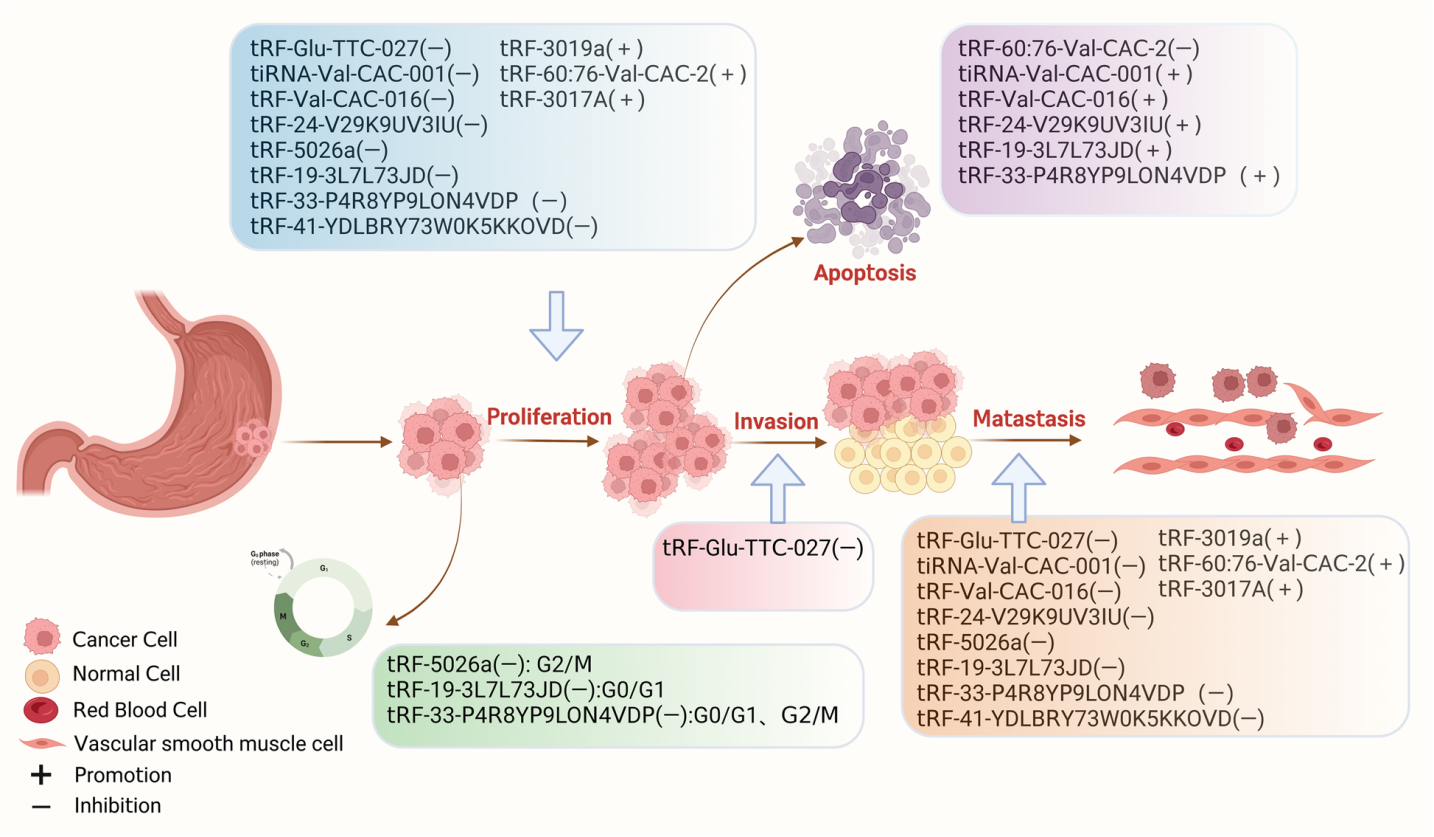
Biofunctions of tsRNAs in GC. tsRNAs can affect the proliferation, invasion, metastasis and apoptosis of gastric cancer cells

The 3rd-peak prevalent driver for tumor-linked fatalities globally is due to gastric cancer with poor prognosis, despite the application of various conventional and modern treatment approaches, including surgery, targeted therapies, chemotherapy, and immunotherapy [55–57]. Currently, tsRNAs are being increasingly investigated for their potential as novel diagnostic/prognostic biomarkers or therapeutic targets in GC. Dysregulating tsRNAs can either promote or inhibit GC development and progression through various mechanistic functions. As shown in Fig. 2, the biological functions of tsRNAs in GC were summarised.

### Biofunctions of tsRNAs in GC

#### Promoting biofunctions of tsRNAs

In numerous ways, tsRNA can enhance GC progression. tRF-Val, which is abundant in GC tissues, directly binds to eukaryotic translation elongation factor 1 alpha 1 (EEF1A1) protein to promote its translocation to the nucleus, improves interactions between EEF1A1 and MDM2 proteins, and promotes tumor protein 53 (p53) ubiquitination, all of which contribute to GC progression [58]. tsRNAs, in addition to RNA-binding protein (RBP) machinery, can play a regulatory role via binding to target genes. In GC cells and tissues, tRF-3019a is highly overexpressed [59]. It can interact with Argonaute 2 (AGO2) protein to target and control F-box protein 47 (*FBXO47*) gene expression, which improves tumor cell proliferation/metastasis. Furthermore, the significantly increased expression of tRF-3017A in GC tissues may increase GC malignancy by blocking the new epidermal growth factor-like-like 2 (*NELL2*) gene [60]. Notably, the different mechanisms of tsRNAs in GC promotion may provide various prospectives in clinical application.

#### Inhibiting biofunctions of tsRNAs

By RNA sequencing, Shen et al. found that tRF-33-P4R8YP9LON4VDP (tRF-33) was significantly reduced in GC tissues. Cell function experiments showed that tRF-33 could inhibit the proliferation, migration, and cycle progression of GC cells and could promote apoptosis, suggesting the potential of tsRNA to regulate GC progression [61]. Another study found that tRF-19-3L7L73JD, which was lowly expressed in GC tissues, could block the cell cycle at the G0/G1 phase, inhibit cell proliferation and migration, and suppress malignant progression of cells [62]. Furthermore, Overexpression of tRF-41, also known as tRF-41-YDLBRY73W0K5KKOVD, has been found to have inhibitory effects on the migration and proliferation of tumor cells. Additionally, it can encourage apoptosis through down-regulating for phosphoadenosine-phosphosulfate synthetase 2 (PAPSS2) [63]. It's worth noting that tsRNAs can modulate relevant signaling pathways to influence the malignant progression of GC. The tRF-Glu-TTC-027 molecule is notably down-regulated within GC tissue, primarily within cytoplasm. The tRF-Glu-TTC-027 molecule can hinder the expression of proteins related to the mitogen-activated protein kinase (MAPK) pathway, which in turn hinders the progression of GC [32]. Another investigation demonstrated that tRF-5026a can drive G2/M cell cycle arrest and impede the GC progressionthrough PI3K/AKT (Phosphoinositide-3 kinase) signaling pathway [64]. tiRNA-Val-CAC-001 by zhang et al. was found to inhibit the proliferation and metastasis of GC cells by regulating the Wnt/β-catenin signaling pathway, which in turn impedes GC progression [65]. Similarly, Dong et al. found that tRF-24-V29K9UV3IU plays a cancer inhibitory role in GC progression by inhibiting the Wnt signaling pathway [66, 67]. According to investigations, tRF-Val-CAC-016 is considerably down-regulated within GC tissue. This down-regulation can effectively hinder the progression of cancer cells. Further research has revealed how the MAPK pathway can be suppressed through tRF-Val-CAC-016 through binding onto Calcium Voltage-Gated Channel Subunit Alpha1 D (CACNA1d) gene, regulating its expression [68]. In conclusion, tsRNAs can regulate the proliferation and metastasis of GC cells and regulate the malignant progression of GC through multiple signaling pathways, providing new insights and targets for the diagnosis and treatment of GC.

### Clinical application of tsRNAs in GC

#### Diagnostic potential for tsRNAs

Several investigations have demonstrated that tsRNAs had a promising diagnostic potential for GC. Compared to patients with gastritis and healthy individuals, GC patients had considerably higher serum expression levels of tRF-23-Q99P9P9NDD [15] and tRF-17-WS7K092 [69]. Interestingly, the expression of them were significantly reduced in GC patients after surgery. tRF-23-Q99P9P9NDD gave an area under the curve (AUC) of 0.783 with a sensitivity of 63% and a specificity of 86% in diagonosis, which was higher than the AUCs of traditional markers, such as carbohydrate antigen 724 (CA724), carbohydrate antigen 199 (CA199), and carcinoembryonic antigen (CEA). However, the AUC was even higher at 0.862 when the four markers were used together to detect gastric cancer. Furthermore, the AUC of tRF-23-Q99P9P9NDD was 0.724 with a sensitivity of 60% and a specificity of 85% for stage I/II GC, which was also superior to the CEA, CA199, and CA724. In addition, the combined AUC was 0.819 [15]. Similarly, tRF-17-WS7K092 showed an AUC of 0.819 in discriminating the GC from healthy individuals by ROC analysis, which was also higher than the AUC of CA724, CA199, and CEA. When all four markers were combined, the AUC increased to 0.882. Another tsRNA, situated on chromosome 6q24.2 and derived from tRNA-Gln-TTG, named as Hsa_tsr016141 was also up-regulated in serum and cancerous tissues of GC, the AUC value of this tsRNA was 0.692, slightly higher than CEA (0.654) and CA199 (0.621) [70]. These results suggest that some highly expressed tsRNAs may be reliable diagnostic markers for GC.

Contrastly, tRF-33-P4R8YP9LON4VDP (tRF-33) and tRF-27-87R8WP9N1E5 (tRF-27) were expressed at significantly lower levels in the plasma of GC patients. Their levels were marked increased in the plasma of GC patients after surgery [71, 72]. The tRF-33 showed an AUC of 0.757 in accurately discriminating earlier GC from healthy individuals [71]. In addition, the AUC for determining advanced GC from healthy individuals was 0.766, while the AUC values for early and advanced GC in gastritis patients were 0.717 and 0.635, respectively. The level of tiRNA-5034-GluTTC-2 was notably lower in both tissue and plasma from GC patients, with the AUC was 0.835 and 0.779, respectively. When the results were combined, AUC was 0.915 [73]. Besides, the AUC of tRF-19-3L7L73JD was 0.6230 in plasma [62], and the AUC of tRF-5026a was 0.908 in GC tissues by the ROC analyses [64]. Another study revealed that the AUC of tRF-3019a in distinguishing healthy tissue from GC with stage I-II, stage III-IV, lymph node metastases, together with low pathological differentiation was 0.796, 0.665, 0.530, and 0.677, respectively [59]. These studies implied that these low expressed tsRNAs exhibited effective application in the diagnosis of GC. Combined ROC analysis of the up- and down-regulated tsRNAs would make it possible to obtain an ideal panel of diagnostic markers.

#### Prognostic value of tsRNAs

Growing evidence have indicated that tsRNAs exhibit favourable prognostic value in GC. The expression of tRF-23-Q99P9P9NDD in serum was closely associated with advanced T stage, TNM stage, lymph node metastasis, and neurovascular invasion in GC patients. Furthermore, Kaplan–Meier curves demonstrated that GC patients who had low level of tRF-23-Q99P9P9NDD had a longer overall survival time [15]. Another study reported that there was a favorable correlation between tRF-17-WS7K092 expressionlevel within GC patient serum together with lymph node status, TNM stage, and neurovascular invasion. Survival analysis showed that patients with low expression of this tsRNA had a good survival outcomes [69]. Downregulation of tRF-33 had been proved to be positively correlated with poor prognosis in advanced GC patients, with lower serum tRF-33 associated with poorer survival [71]. Patients with high levels of tRF-27 had larger tumors, higher Ki67 levels, and shorter survival. Moreover, COX analysis demonstrated that tRF-27 was an independent predictor for GC patients. tRF-31-U5YKFN8DYDZDD (tRF-31) was considerably upregulated in GC tissue, and was positively associated with tumour size before surgery. Patients with high levels of tRF-31 were had poorer survival compared to those with low levels. COX multivariate analysis revealed that tRF-31 was a standalone predictor, demonstrating its strong predictive value for the risk of death from GC [74]. The downregulation of tRF-5026a is associated with larger tumors, later TNM stage, and metastasis to additional lymph nodes in GC patients, and is considered an independent prognostic marker by COX regression. In addition, the low level group had a longer survival time compared to patients with high levels of tRF-5026a [64]. Besides, hsa_tsr016141 was correlation to the reduced degree of differentiation, later T stage, and positive lymph node metastases. GC patients had increased expression of hsa_tsr016141 had a worse prognosis [70]. These findings suggest that they may have potential prognostic significance for GC individuals.

#### tsRNAs within CRC

**Fig. 3:**
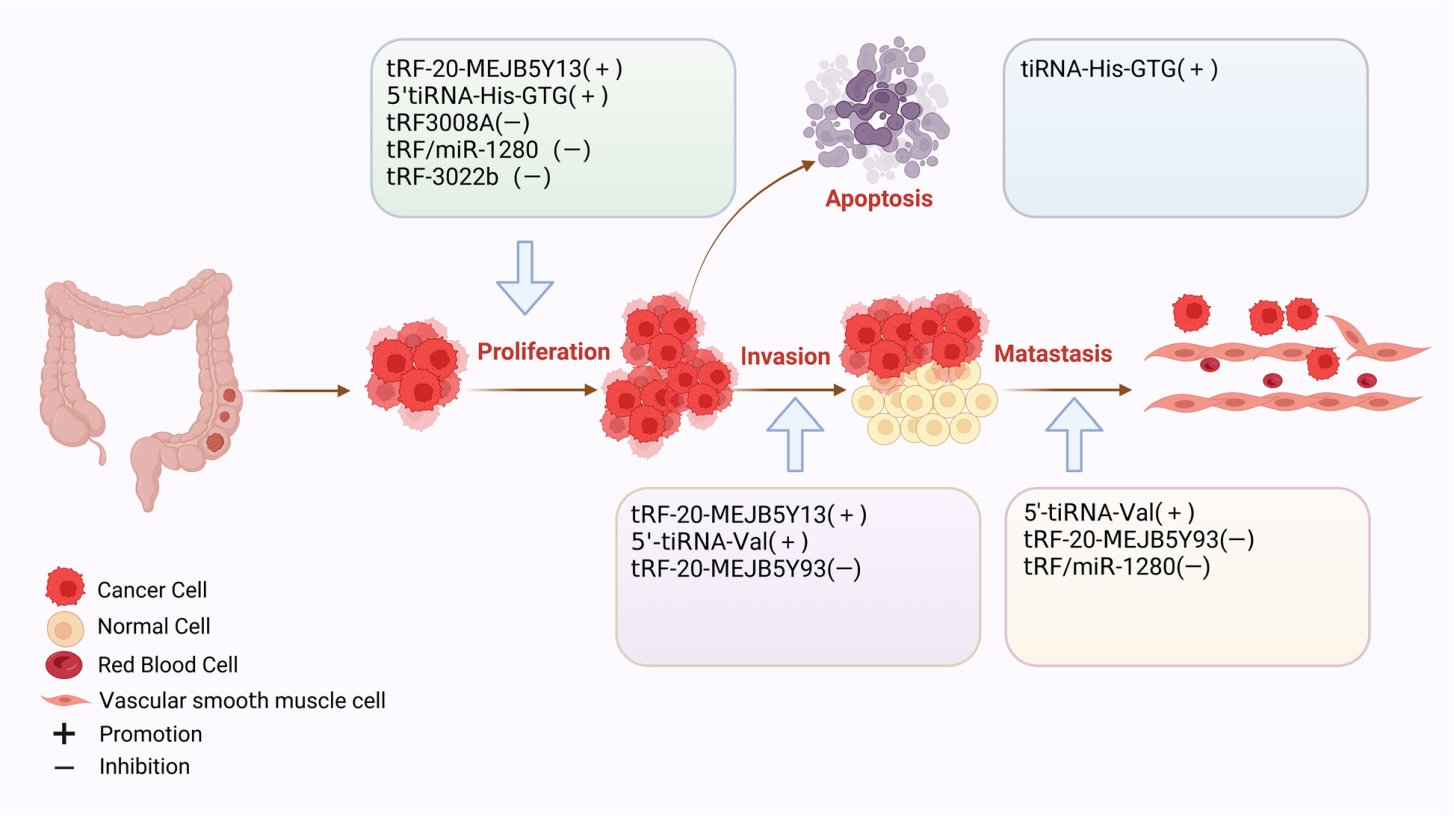
Biofunctions of tsRNAs in CRC. tsRNAs can affect the proliferation, invasion, metastasis and apoptosis of colorectal cancer cells

CRC has the third highest mortality rate of all cancers. There were a significant increase in new cases and deaths [22]. Growing evidence points to the biological significance of some tsRNAs in the development of CRC. As shown in Fig. 3, the biological functions of tsRNAs in CRC were summarised.

### Biofunctions of tsRNAs in CRC

#### Promoting biofunctions of tsRNAs

Studies had reported some tsRNAs might act as carcinogenic molecules in CRC. Xiong et al. identified 16 abnormal tsRNAs by RNA sequencing in CRC. Functional enrichment revealed that tRF-25-P940KK5Y93, tRF-24-NMEH623K25, tRF-30-XSXMSL73VL4Y, tRF-26-P4R8YP9LOND, and tRF-29-P27JPJ60MVJY might facilitat the progression of CRC through vitamin metabolism pathways, while tRF-29-QU7BPN6ISBJO and tRF-27-Q99P9P9NH5N accelerate the development of CRC via the cGMP-PKG pathway [16]. Another tsRNA, named as tRF-20-MEJB5Y13, derived from Dicer1 (a type of ribonuclease), promotes the CRC proliferation and metastasis through the epithelial-to-mesenchymal transition (EMT) pathway [33]. Furthermore, one derivative of tRNA-Val, called 5'-tiRNA-Val, is up-regulated in CRC tissues and accelerates invasion and metastasis but does not affect proliferation of CRC cells [75]. 5'-tiRNA-His-GTG has a length of 35 nt and was primarily found in cytoplasm. It is significantly increased in CRC tissues and silenced the large tumor suppressor kinase 2 (*LATS2)* with the complementary role of AGO, and then promotes the progression of CRC by inhibiting the Hippo signalling pathway [76]. More in-depth studies of these tsRNAs may provide new targets for CRC therapy.

#### Inhibitory biofunctions of tsRNAs

Some down-regulated tsRNAs have been reported to act as oncogenic molecules in the development of CRC. Luan et al. found that tRF-20-MEJB5Y93 could suppress the CRC metastasis by targeting claudin-1 and interfering with the EMT [77]. Furthermore, they confirmed that it could bind to the 4957–4976 region and silence the expression of MALAT-1, which could regulate alternative splicing of structural maintenance of chromosomes 1A (SMC1A) by interacting with serine and arginine rich splicing factor 2 (SRSF2), leading to a block in CRC metastasis [78]. tRF3008A, which is derived from 3′-tRNA^val^, cloud bind to the Argonaute related complex and reduce the stability of *FOXK1*, resulting in down-regulation of *FOXK1*, obstruction of Wnt/β-catenin signaling pathway, and inhibition of CRC progression [79]. tRF/miR-1280 is produced by tRNA^Leu^ and pre-microRNA, it can combin the 3'UTR region of *JAG2* and inhibite its expression, and then regulate the Notch signalling pathway, and finally reduce the metastasis and formation of CRC; In addition, inactivation of Notch signalling pathway suppressed cancer stem-like cells (CSC) phenotypes, such as direct transcriptional silencing of the Gata1/3 and miR-200b [80]. Yang et al.reported that tiRNA-His-GTG could promote the increase of apoptosis but does not affect the cellular viability [81]. tRF-3022b candirectly target LGALS1 and macrophage migration inhibitory factor (MIF), resulting in suppression of M2 macrophage polarisation and CRC progression [82]. tRNA-Val (UAC) and tRNA-Leu (CAG) extracted from *Escherichia coli* are cytotoxic and can significantly inhibit the clonogenic ability of CRC cells [83, 84]. These findings revealed inhibitory biofunctions of some tsRNAs, which are expected to be novel potential therapeutic targets for CRC.

### Clinical application of tsRNAs in CRC

#### Diagnostic value of tsRNAs

Recently, more and more tsRNAs have been found to be aberrantly expressed in CRC samples, providing potential diagnostic value for these patients. tRF-18-8R1546D2, tRF-22-WE8S68L528R1546D2, tRF-22-WB86Q3P92, and tRF-22-WE8SPOX52 had been identified anomaly expressed in CRC tissues by Zhu et al. and were used to further develop a novel diagnostic model. ROC analysis showed an AUC value of 0.93 [85]. 5'-tRF-GlyGCC is a 31nt fragment, a highly abundant tsRNA in human fluids, and is considerably up-regulated in the plasma of CRC patients [86]. The AUC for 5'-tRF-Gly^GCC^ was 0.882, significantly higher than CEA (0.762) and CA199 (0.557); the AUC (0.926) for the combined diagnosis was the highest [86]. tRF-VAL-TCA-002 and tRF-phe-GAA031 were obviously upregulated in CRC tissues, with AUC of 0.7554 and 0.7313 respectively in diagnosis [87]. tRF-3022b, derived from tRNA-Gly, is overexpressed in CRC tissues. The AUC of tRF-3022b in separating CRC patients from healthy subjects was 0.7684 in plasma samples, while was 0.8266 in tissues, suggesting a strong diagnostic value of minimally invasive [82]. Although these tsRNAs have severally excellent value in the diagnosis of CRC, a better diagnostic effect is expectable based on the combined analysis of these tsRNAs.

#### Prognostic value of tsRNAs

tsRNAs have shown potential value in the prognosis of CRC. Zhu et al. constructed a prediction model based on tRF-33-PSQP4PW3FJI0W, tRF-18-HSRVK7D2, tRF-18-H9Q867D2, tRF-33-PSQP4PW3FJIKW, tRF-16-I3FJQSD, and tRF-32-O7M8LOMLQHWU3 in CRC. Patients with a high-risk score had a significantly shorter survival time. Multivariate analysis confirmed that this prognostic model was an independent indicator in CRC patients [85]. 5'-tiRNA-Pro^TGG^ is down-regulated and an independent prognostic factor in CRC. Patients with positive expression of 5'-tiRNA-Pro^TGG^ have significantly shorter disease-free survival (DFS), increased tumor recurrence rate, and shorter overall survival than those with negative expression [88]. tRF-phe-GAA031 and tRF-VAL-TCA-002 are significantly negatively correlated with the overall survival rate, proving that they may be excellent prognostic biomarkers in CRC [87]. Besides, the serum level of 5'-tiRNA-Val in CRC patients is considerably higher than healthy controls, and its expression is positively associated with tumor lymph node metastases, which can be used as a biomarker to assess the status of lymph node metastasis [75]. Overall, these tsRNAs have promising applications in the prognosis of CRC patients.

#### tsRNAs in hepatobiliary cancers

**Fig. 4:**
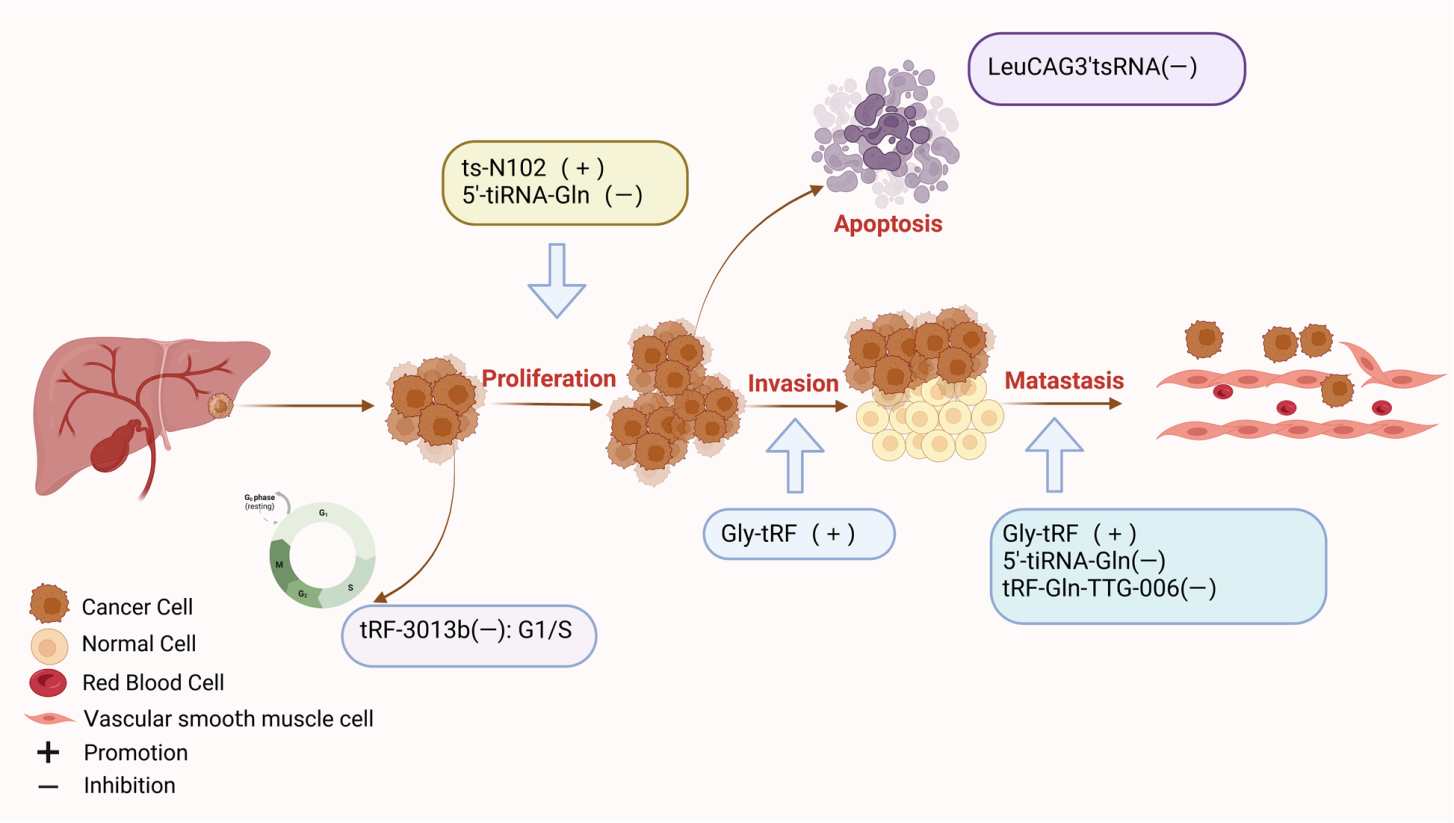
Biofunctions of tsRNAs in Hepatobiliary Cancers. tsRNAs can affect the proliferation, invasion, metastasis and apoptosis of hepatobiliary cancer cells

Hepatobiliary cancer includes GBC, CCA, and HCC [89]. Although the survival has improved with systemic immunotherapy and targeted therapy, the prognosis for advanced patients is unsatisfactory [90]. Thus, search for novel and effective biomarkers is particularly important for hepatobiliary carcinoma. As shown in Fig. 4, the biological functions of tsRNAs in hepatobiliary cancers were summarised.

### Biofunctions of tsRNAs in hepatobiliary cancers

#### Promoting biofunctions of tsRNAs

Unlike GC and CRC, the role of tsRNAs in hepatobiliary cancer is not well understood. However, some studies have reported that tsRNA plays an important role in promoting the progression of hepatocellular carcinoma. LeuCAG3'tsRNA, 22 nt length, is highly expressed in mouse model of hepatobiliary cancer, which can regulate the translation of RPS28 and reduce apoptosis [91]. Gly-tRF is upregulated in hepatobiliary cancer cells and promotes EMT of hepatic tumor stem cells. Specifically, Gly-tRF can directly target the Nedd4 family-interacting protein 2 (NDFIP2), which is a component of the AKT signalling pathway, leading to migration and EMT [92]. Another study discovered that 5’tRF-Gly can bind and silence the expression of carcinoembryonic antigen-related cell adhesion molecule 1 (*CEACAM1*), and then promote the proliferation, migration, and invasion of hepatobiliary cancer cells [93]. Analysis revealed ts-N102 may inhibit the expression of hs-mir-215 and act as a proto-oncogene in liver cancers. Functional enrichment analysis indicated that ts-N102 may be involved in fatty acid synthesis and metabolism [94].

#### Inhibitory biofunctions of tsRNAs

The living organism is like a highly complex machine, inhibitory biofunctions of tsRNAs also exist to maintain the homeostasis of life. 5'-tiRNA-Gln, produced from tRNA^Gln−TTG^, is over-expressed in hepatobiliary cancers. It can interact with eukaryotic translation initiation factor (EIF4A1) after forming a G-quadruplex structure, and then inhibit proliferation and migration of hepatobiliary cancer cells [95]. tRF-3013b is down-regulated and inhibits the progression of GBC. It directly targets the tumor protein P63 regulated 1 like (*TPRG1L*) and then downregulates NF-κB, c-myc, and CDK2, resulting in GBC cells remaining in G1/S phase [96]. tRF-Gln-TTG-006 has been shown to have a tumor suppressive effect but the mechanism is still unknown [97].

In addition, some tsRNAs, whose promoting or inhibiting effects have not been clearly defined, also play a crucial role in hepatobiliary cancers. For instance, Li et al. identified many differentially expressed tsRNAs in CCA and selected tRF-34-JJ6RRNLIK898HR, tRF-38-0668K87SERM492V, and tRF-39-0668K87SERM492E2 with statistical significance for further evaluation. These tsRNAs are both derived from tRF^GluTTC^ and can target the Ruppel-like factor family16 (*KIF16*) to regulate fatty acid formation and influence CCA progression [98].

### Clinical application of tsRNAs in hepatobiliary cancers

Aberrantly expressed tsRNAs in hepatobiliary cancer also have potential value for clinical application. The expression of tRNA-ValTAC-3, tRNAGlyTCC-5, tRNA-ValAAC-5, and tRNA-GluCTC-5 in the plasma of patients was significantly higher than in healthy controls, suggesting a minimally invasive diagnostic value in hepatobiliary cancer [34]. The level of tRF-Gln-TTG-006 was significantly increased in the serum of HCC patients. ROC analysis were performed to discriminate HCC patients from healthy controls in two cohorts from different hospitals, and the AUC was 0.919 and 0.875, respectively. While combined two cohorts, the AUC was 0.875. These results indicated that there is a high individual heterogeneity in the level of tRF-Gln-TTG-006 in HCC patients. In addition, further evaluation revealed that the AUC of tRF-Gln-TTG-006 was 0.858, which was better than AFP in the differentiation stage I, indicating that it is an effective biomarker for HCC [97]. 5'tRF-Gly is upregulated in hepatobiliary cancer tissues and cells, and positively correlates with tumor growth, distant metastasis, and poor differentiation. Individuals who expressed more 5'tRF-Gly had shorter survival times [93]. ts-N102 was found to be upregulated in hepatobiliary cancer tissues, while ts-N7, ts-N94, ts-N84, and ts-N37 were down-regulated. To distinguish primary tumors from healthy tumors, The ROC analysis showed a AUC value of 0.88 in diagnostic effect based on these five tsRNAs. In addition, seven tsRNAs (ts-N20, ts-N21, ts-N22, ts-N36, ts-N37, ts-N44, and ts-N64) were used to construct a risk model, which was proved to accurately predict the prognosis for hepatobiliary cancer [94]. These findings suggest that these up- or down- regulated tsRNAs may have potential application in the diagnosis and prognosis of hepatobiliary cancer.

#### tsRNAs in other cancers

**Fig. 5:**
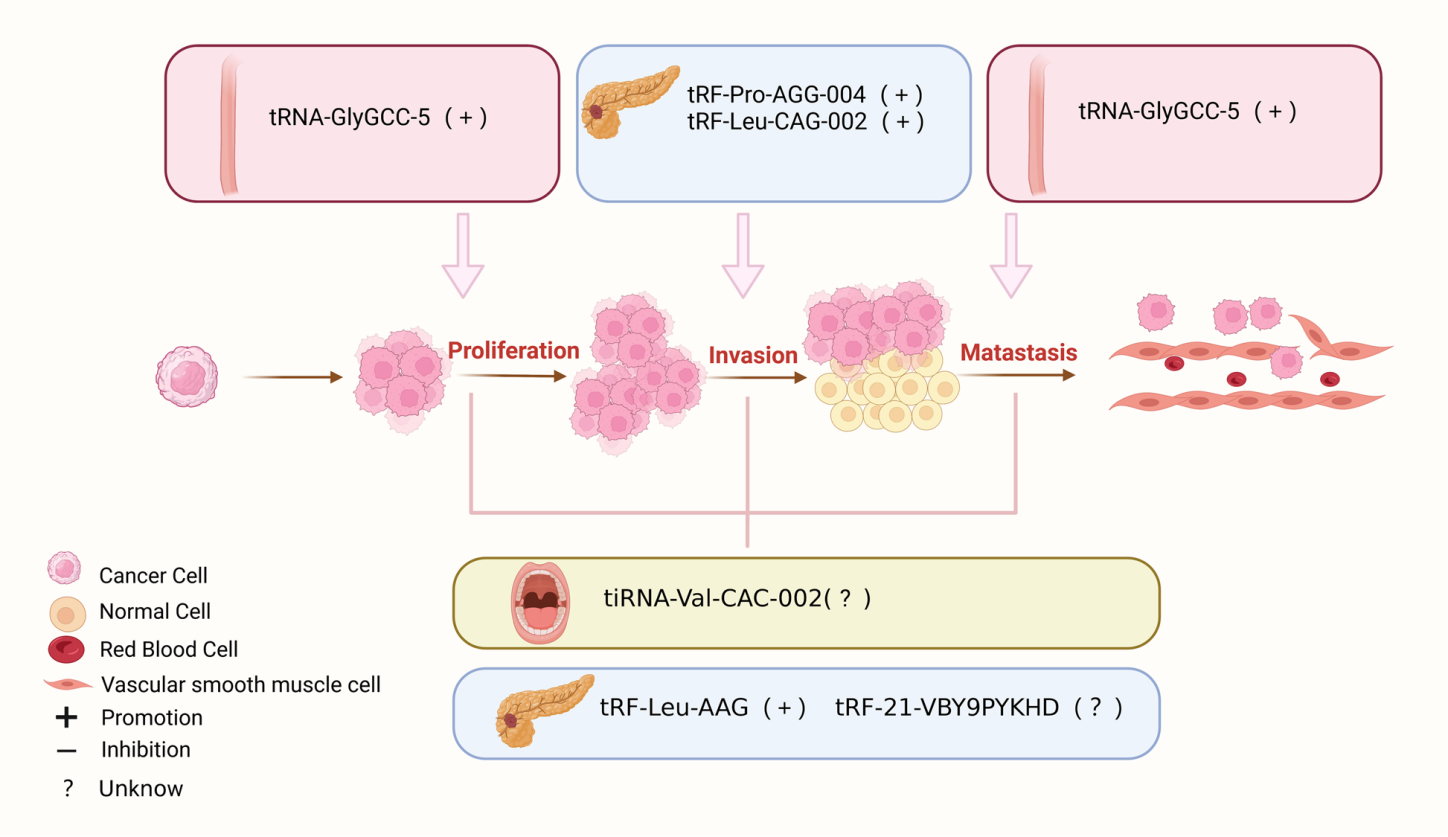
Biofunctions of tsRNAs in Other Cancers. tsRNAs can affect the proliferation, invasion, metastasis and apoptosis of OSCC, EC, and PC cells

tsRNAs also participate in the development and progression of other digestive system cancers, including oral, hypopharyngeal, esophageal, and PC. As shown in Fig. 5, the biological functions of tsRNAs in these cancers are summarised.

### Biofunctions of tsRNAs in other cancers

tiRNA-Val-CAC-002 is a member of the 5'-tRF family and is located on chromosome 1. It is weakly expressed in oral squamous cell carcinoma (OSCC) tissues and may contribute to OSCC development via the PI3K/AKT pathway [17]. tRF-21-VBY9PYKHD (tRF-21), derived from tRNA^GlyGCC^, is a tumor suppressor in PC. Reduced tRF-21 promoted AKT2/1-mediated heterogeneous nuclear ribonucleoprotein L (hnRNP L) phosphorylation, and then phosphorylated hnRNP L enhanced interaction with hnRNP L-DEAD-box helicase 17 (DDX17) and formed a complex of alternative splicing, promoting PC cell malignancy. Furthermore, treatment with tRF-21 mimics could suppressed tumor growth and metastasis in PC mouse xenografts, suggesting that tRF-21 may serve as a therapeutic target to inhibit PC progression [99]. tRF-Leu-AAG can directly target and inhibit the expression of Up-frameshift proteins (UPF1). Knockdown of tRF-Leu-AAG could suppress the the proliferation, metastasis, and invasion of PC cells [100]. tRF-Leu-CAG-002 and tRF-Pro-AGG-004 could promote PC cell migration and tumor growth [101]. Previously study had shown that increased expression of tRNA-GlyGCC-5, formed through 5'end of TRG-GCC, promotes the proliferation and metastasis in EC [31].

### Clinical application of tsRNAs in other cancers

tRF-20-S998LO9D, derived from tRNA86^ArgTCT^, is significantly up-regulated in squamous cell carcinomaof the oral tongue (SCCOT). Individuals with high levels of tRF-20-S998LO9D had shorter survival times and the prognositic AUC value is 0.669 [102].

tRF-1:30-Lys-CTT-1-M2 was highly expressed in hypopharyngeal cancer and correlated with a later tumor stage and poorer differentiation. ROC analysis showed that it had an excellent AUC of 0.9172, indicating a good diagnostic value. Furthermore, it was observed as a unique risk factor for the lung metastasis by COX multivariate analysis [30]. tRNA-GlyGCC-5 had an AUC of 0.878 for discriminating EC patients from healthy individuals, indicating the potential diagnostic role of this tsRNA [31].

tRF-Leu-CAG-002 and tRF-Pro-AGG-004 were significantly elevated in the blood of PC patients. Their AUC values in identifying PC patients from healthy individuals were 0.9 and 0.78. The combined diagnostic efficacy was significantly better than CEA and CA19-9 in differentiating stage I/II patients. Poorer survival rates in patients with high level of these tsRNAs can be employed as markers for post-operative tumor monitoring [101]. tsRNA-MetCAT-37 and tsRNA-Val-TAC-41 are considerably elevated in the tissues and serum of PC patients. The AUC values of them in combination with CA199 were 0.949 and 0.947, respectively, indicating good diagnostic value in distinguishing PC from healthy populations. In addition, the upregulated of tsRNA-ValTAC-41 was positively associated with later tumor stage and distant metastasis [103]. tRF-Pro-CGG was downregulated in PC tissue, and its AUC was 0.92 for identifying PC patients from healthy controls. Low expression of tRF-Pro-CGG was associated with positive lymph node metastasis and later tumor stage. Kaplan–Meier analysis showed that PC patients with low levels of tRF-Pro-CGG had a shorter overall survival times, suggesting its prognostic and diagnostic value [35]. In addition, AS-tDR-000064, AS-tDR-000069, AS-tDR-000102, and AS-tDR-001391 were found to be differentially expressed in PC. However, further studies are required to confirm these clinical application value [104].

**Fig. 6:**
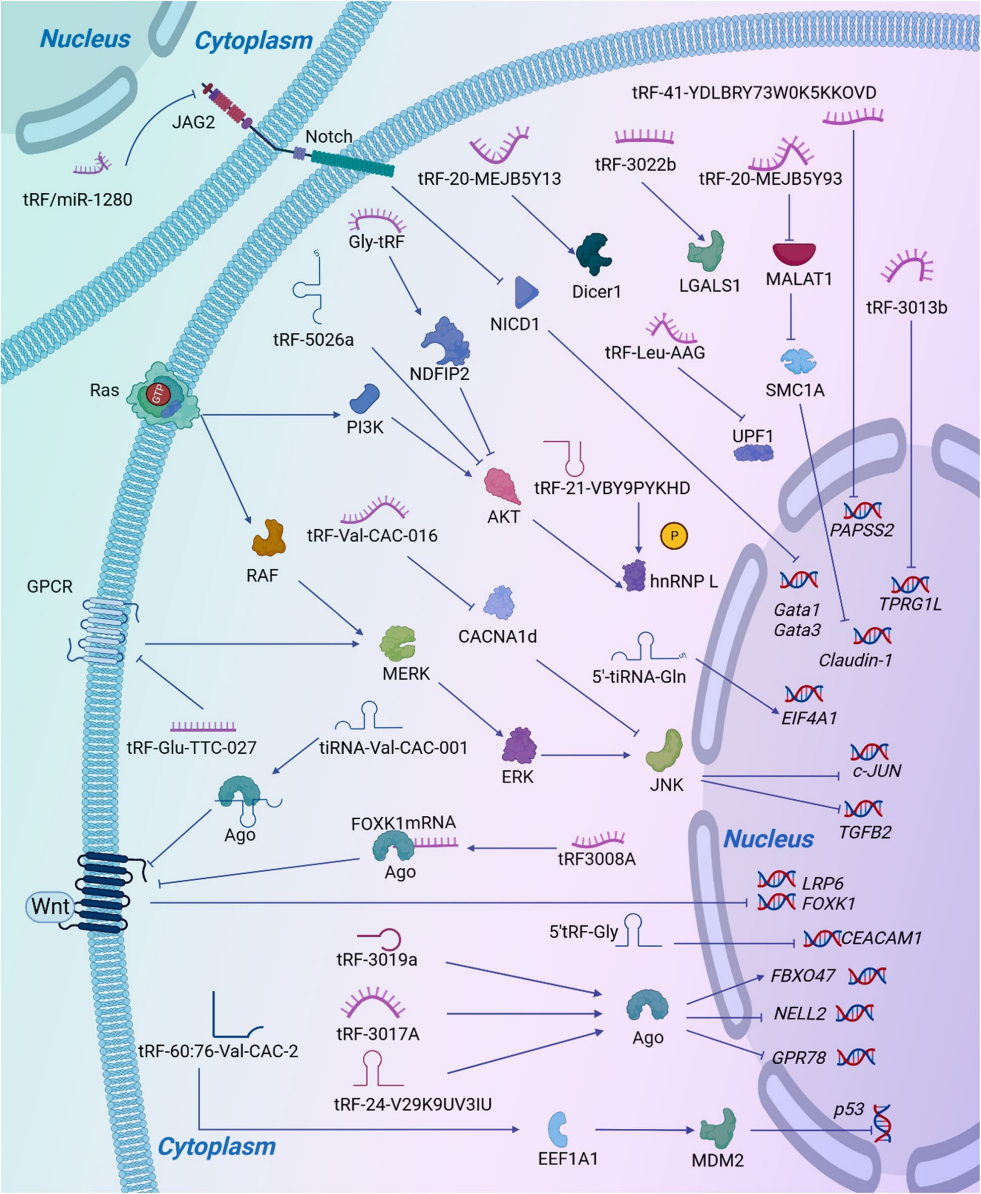
Regulatory mechanism of tsRNAs in cancer cell. tsRNAs interact with proteins and genes to display biological functions in digestive system cancers

In a word, tsRNAs interact with specific genes and proteins to display carcinogenic and inhibitory functions in digestive system cancers (Fig. 6). Our review may provide a better perspective on the function and clinical application prospects of tsRNA in digestive system cancers.

### tsRNAs in benign digestive system diseases

tsRNAs also have pivotal roles in benign digestive system diseases. The association between tsRNAs and oral submucous fibrosis (OSF), oral flora disorder, irritable bowel syndrome (IBS), benign liver diseases, and acute pancreatitis (AP) will be discussed in this section (Table 1).

**Table 1:** The expression, clinical value and functions of tsRNAs in benign digestive system diseases

Disease	tsRNA	Expression	Function	Clinical value	Ref
**OSF**	Gly-TCC-016	Up	Promotion	Treatment	[17]
**IBS**	tiRNA-HIs-GTG-001 tRF-Ser-GCT-113 tRF-Gln-TTG-035	Up Down Down	**-** **-** **-**	Diagnosis\Treatment Diagnosis\Treatment Diagnosis\Treatment	[105]
**Viral hepatitis**	5'tRH^Val^ 5'tRH^Gly^	Up Up	**-** **-**	- -	[106]
**Liver injury**	tRF-Gln-CTG-026	Up	Inhibition	Treatment	[107]
**ACLF**	tsRNA-20 tsRNA-46	Down	- -	Diagnosis Diagnosis	[108]
**AFLD**	Gly-tRFs	Up	Promotion	Treatment	[40]
**NAFLD**	tRF-47-58ZZJQJYSWRYVMMV5BO tRF-3001b	Up Up	Inhibition Promotion	Treatment Treatment	[109, 110]
	tRF-Val-CAC-005 tiRNA-His-GTG-001 tRF-Ala-CGC-006	Up	- - -	Diagnosis\Treatment Diagnosis\Treatment Diagnosis\Treatment	[111]
**AP**	tRF3-Thr-AGT	Down	Inhibition	Treatment	[18]

#### Benign oral disease

##### ***OSF***

OSF is a precancerous disease in which 1.5–15% can become oral malignancies [112]. Therefore, developing and applying molecular biomarkers for the treatment and prevention of OSF is particularly important. Investigations revealed that tRF-Gly-TCC-016 is upregulated in OSF tissue, often targeting IL-13 and TGF-β family cytokines and influencing OSF formation through cytokine‒cytokine receptor interactions and cAMP signaling; tRF-Gly-TCC-016 is deemed a molecular marker and therapeutic target for OSF [17]. Conversely, tiRNA-Val-CAC-002 is down-regulated in tissues. The target genes of tiRNA-Val-CAC-002 are predominantly enriched in membranes and organelles, their primary roles are centered around the ATP metabolic and the modulation of biological reactions to amyloid beta [17].

##### ***Oral flora disorder***

Fusobacterium nucleatum (Fn) is essential in treating oral disorders [38]. He et al. discovered that some tsRNAs could target infections, inhibit ribosome targeting in Fn, and regulate the microbe-host environment [113, 114]. They found tsRNA-000794 and tsRNA-020498 can limit Fn growth, by preventing amino acid synthesis of proteins [113]. More interestingly, NOKSI (the normal oral keratinocyte) cells infected with Fn produced more tsRNA-000794 and tsRNA-020498 at specific time points [113]. This implies that there is a potential interaction between oral keratinocytes and oral bacteria mediated by specific tsRNAs. However, this study did not explore the mechanism by which tsRNAs interact with bacteria, which is crucial for studying the relationship between oral diseases and microbiota.

#### IBS

Irritable bowel syndrome with diarrhea (IBS-D) is a functional bowel disorder [115], but much of its pathogenesis is unknown [116]. tiRNA-His-GTG-001, tRF-Ser-GCT-113, and tRF-Gln-TTG-035 have been linked to functional intestinal disorders in investigations [105]. tiRNA-His-GTG-001 was found located on chromosome 9 at gene tRNA-His. The length of tiRNA-His-GTG-001 is 34 nt, which is derived from the 5' end of tRNA-His-GTG and is highly expressed in IBS patients. Biological function analysis revealed that the target genes of tiRNA-His-GTG-001 exhibit significant associations with crucial pathways and metabolic processes, including but not limited to glutamate metabolism, GABAergic synapse, TNF-α signaling pathway, and insulin resistance. Analysis showed that tiRNA-His-GTG-001 can target GABAB receptor 2 (GABBR2), which is a major nociception molecule that plays an important function in pain regulation [117]. Thus, tiRNA-His-GTG-001 may modulate irritable abdominal pain in irritable bowel syndrome by targeting GABABR2 with its 3′UTR.The length of tRF-Ser-GCT-113 is 31nt and is low expressed within intestinal mucosa of irritable bowel patients, which is found on chromosome M at the gene MT-TS2. tRF-Ser-GCT-113 is associated with some symptoms of irritable bowel, such as bloating and diarrhea. 5-hydroxytryptamine 2C receptor (HTR2C) and SAM and SH3 domaincontaining protein 1 (SASH1) were the genes targeted by tRF-Ser-GCT-113, which were associated with stress sensitivity and clinical mood disorders [118], suggesting that tRF-Ser-GCT-113 may trigger patients to develop abdominal discomfort, bloating, and other symptoms by acting on HTR2C and SASH1. While length of tRF-Gln-TTG-035 is 14 nt, low expressed in intestinal mucosa of IBS patients. The target genes associated with tRF-Gln-TTG-035 are linked to the PPAR signaling pathway. It can bind to and act on Toll-like receptor 4 (TLR4), and gamma-aminobutyric acid receptor-associated protein (GABARAP) to inhibit their translation. Additional research indicated that tRF-Gln-TTG-035 influenced immune-mediated signaling in the brain-intestinal neurons by modulating TLR4 and GABARAP, resulting in an increased extent of dilation of the abdomen. The ROC analysis conducted on the patient group and the normal control group revealed that the AUC values of tiRNA-His-GTG-001, tRF-Ser-GCT-113, and tRF-Gln-TTG-035 were 0.921, 0.792, and 0.889, respectively, indicating that they might have potential as effective diagnostic markers and new therapeutic targets [105].

#### Benign liver disease

Liver injury and failure are common life-threatening diseases requiring multidisciplinary and collaborative treatment [39, 119].

##### ***Liver injury***

tRF-Gln-CTG-026 belongs to tRF-1, whichinteracts with TSR1 (pre-rRNA processing protein TSR1 homolog) to inhibit TSR1 and 18S rRNA action, consequently decreasing ribosome assembly, reducing global protein synthesis, and alleviating liver injury, indicating that it is a possible therapeutic target and it can offer a novel approach for addressing liver injury [107].

##### ***Viral hepatitis***

Until now, the prevalence of HBV (hepatitis B virus) remained elevated [120], with an estimated 350 million individuals worldwide living with chronic HBV infection, while more than 160 million people were chronically infected with hepatitis C virus (HCV). Tragically, over one million patients succumbed to complications linked to chronic viral hepatitis during this period [121]. 5'tRHs (5'-tiRNA) with a length of 30–35 nucleotides play a crucial role during these viral infection processes [106]. The researchers found that the expression of 5'tRH^Val^ and 5'tRH^Gly^ were significantly increased in liver tissue infected with HBV and HCV. Furthermore, their research group discovered that 5'tRH^Val^ and 5'tRH^Gly^ do not participate in the control of protein synthesis in hepatoma cells. The role of 5'tRHs in viral hepatitis is worthy of further investigation [106].

##### ***Liver failure***

The expression of tsRNA-20 and tsRNA-46 is downregulated in HBV associated acute, chronic liver failure (ACLF) [108]. Biological functions analysis demonstrated a significant correlation between them with the MAPK signaling pathway, the hepatitis B pathway and other pathways related to metabolism, proliferation and apoptosis. A MTR-RNA signature, including tsRNA-20 and tsRNA-46, along with other ncRNAs, were constructed to detect ACLF. This MTR-RNA signature could proficiently differentiate HBV-ACLF cases from the normal controls. The researchers divided the cohorts into the training group and the verification groups. In the training cohort, the signature showed a diagnostic efficiency with an AUC of 0.75 and a sensitivity of 86.25%. In the validation cohort, the MTR-RNA signature effectively differentiated HBV-ACLF cases from normal subjects, achieving an AUC of 0.787, along with a sensitivity of 74.29%. The result suggested that tsRNA-20 and tsRNA-46 might become important indicators in disease diagnosis [108].

##### ***Fatty liver***

Fatty liver is a metabolic-related disease; no effective drugs prevent it [122]. Within the investigation of alcoholic fatty liver disease (AFLD), a sequencing analysis revealed that Gly-tRFs, spanning a range of 29–34 nt in length, underwent cleavage at the anticodon loop of their tRNA precursors from the 5′ end. They exhibited a notable upregulation in AFLD patients (mice subjected to chronic ethanol consumption). Further research found that complement C3 can promote the production of Gly-tRFs by modulating the expression of CYP2E1 which is a cytochrome P450 enzyme. Gly-tRFs participate within the sirtuin 1 (SIRT1) signaling pathway to regulate lipid metabolism, and Gly-tRF inhibitors will become a therapeutic strategy for AFLD [40].

In an investigation of nonalcoholic fatty liver disease (NAFLD), the expression of tRF-47-58ZZJQJYSWRYVMMV5BO (tRF-47) was considerably upregulated. Further study found that increased expression of tRF-47 enhanced autophagy, reduced pro-death signals, and reduced lipid deposition, indicating that it can achieve the therapeutic effect of NAFLD by promoting autophagy process [109]. Study showed that tRF-3001b exhibited a markedly increased expression in patients with NAFLD. Further experimental analysis revealed that tRF-3001b inhibits autophagy through the Prkaa1 pathway, leading to the formation of lipids and aggravating the progression of NAFLD. tRF-3001b is elevated in a NAFLD mouse model, and it can inhibit the expression of the autophagy gene *Prkaa1* and exacerbate NAFLD [110]. Hence, silencing tRF-3001b to diminish hepatic lipid formation and consequently ameliorate NAFLD development, which may be a novel approach for NAFLD treatment. Huang et al. observed that a substantial increase in some tRNA fragments in both liver tissues and plasma obtained from individuals diagnosed with NAFLD [111]. Among these, tRF-Ala-CGC-006, tiRNA-His-GTG-001, and tRF-Val-CAC-005 were chosen for comprehensive investigation due to their high levels in expression. The biological function analysis revealed their primary involvement the control of lipid metabolism. Their AUC values for distinguishing between NAFLD and non-NAFLD samples were 0.875, 0.840, and 0.868, respectively. It has been proposed that they have excellent diagnostic value and could be used as biomarkers. Notably, research has confirmed some tRFs like tRF^GluTTC^ could inhibit adipogenesis, concomitant with the downregulation of adipogenic transcription factors such as aP2 and PPARγ, suggesting their important role in lipid metabolism [123].

#### AP

AP is a chronic inflammatory condition becoming more common [124, 125]. In AP animal models, it was discovered that tRF3-Thr-AGT expression was down-regulated. When tRF3-Thr-AGT expression was elevated, several inflammatory factors in AR42J cells (such as caspase1, IL-1β and IL-18) were inhibited, which implies its potential involvement in the initiation of inflammation during pancreatitis. Bioinformatics analysis showed that ZBP1, CD44 and BTG2 were the downstream target of tRF3-Thr-AGT. It had the capability to attach to the 3'UTR of ZBP1 and could influence the onset of cell pyrosis and inflammation via Z-DNA-binding protein 1 (ZBP1) and control the ZBP1/NLRP3 pathway to prevent cell pyrosis and inflammation. These data suggest that tRF3-Thr-AGT has an inhibitory effect on the progression of pancreatitis and is a protective factor for AP [18]. In another research by this team, they built a pancreatic acinar intracellular trypsinogen activation model (PAITA) and found that tRF3-Thr-AGT was the hub tRFs in this model. The level of its expression is notably diminished. tRF3-Thr-AGT is also associated with trypsinogen activation, providing a novel biomarker for diagnosing and treating AP [126].

## Conclusions and prospects

tsRNAs, an emerging class of small noncoding RNA molecules, have many biological roles, including RNA silencing, RNA stability modulation, transcription and reverse transcriptional regulation, and translation regulation [127–131]. High-throughput sequencing, bioinformatics analysis, and cell experiments are the main methods used to explore the relationship between tsRNAs and diseases. tsRNAs are considered promising diagnostic and predictive markers or potential molecular therapeutic targets in many diseases due to their association with an increasing number of disease processes, their influence on the occurrence and progression of disease, and their widespread presence in human blood, urine, and semen.

Some investigations have explored the relationship between tsRNAs and human tumors such as breast, stomach, colorectal, lung, and other malignant tumors [132, 133]. Nonetheless, to the best of our understanding, this is the first systematic review to summarize the biological function and therapeutic application prospects of tsRNAs in digestive system cancer and benign disorders. In this review, we examined the association between tsRNAs and numerous digestive system disorders, including cancer and benign diseases, and outlined the biological activities and therapeutic application prospects of individual tsRNAs. We discovered that existing research still has the following issues: (1) Most investigations have not deeply studied the relationship between tsRNAs and the pathogenesis of diseases, while investigations have focused on the biological function of tsRNAs and their influence on diseases. Few researchers have proposed the regulatory mechanism and network involved in tsRNAs. (2) When judging the diagnostic and prognostic value of tsRNAs, the sample size of some investigations is insufficient, resulting in insignificance of the diagnostic AUC value and predictive analysis results. (3) The expression level of tsRNAs varies greatly in different tissues and cells [134], which is related to cell type, cell state, and function, and whether the expression of tsRNAs changes at different stages for same disease remains to be studied. (4) Although tsRNAs exist in serum, plasma, saliva, and urine, tsRNAs themselves have many modifications [135]. The stability of tsRNAs, their capacity to be recognized through RNase, and ultimately their ability to generate tsRNAs are all impacted by these alterations, which considerably affect the structure and function of tsRNAs [136]. Therefore, whether the different types of tsRNAs produced at different sites can be stably expressed in exosomes as biomarkers still needs to be verified in many investigations.

In summary, research on the function of tsRNAs in the occurrence and progression of digestive system disorders can offer new molecular markers for the early diagnosis, prognosis evaluation, and therapeutic response monitoring of digestive system disorders; these tsRNAs can also serve as new targets in clinical treatment strategies, which is of extreme relevance for enhancing the accuracy of diagnosis and the efficacy of treatment. However, extensive research is still needed before tsRNAs can be applied as molecular markers and therapeutic targets in clinical, disease diagnosis and treatment.

## Data Availability

All data included in this study are available upon request by contact with the corresponding author.

## Abbreviations

tsRNAs: TRNA-derived small RNAs
ncRNAs: Non-coding RNAs
tRNA: Transfer RNA
tRFs: TRNA-derived fragments
pre-tRNAs: Precursor of tRNA
CRC: Colorectal cancer
GC: Gastric cancer
HCC: Hepatocellular carcinoma
EC: Esophageal cancer
PC: Pancreatic cancer
CCA: Cholangiocarcinoma
GBC: Gallbladder cancer
OSCC: Oral squamous cell carcinoma
SCCOT: Squamous cell carcinoma of the oral tongue
OSF: Oral submucous fibrosis
IBS-D: Irritable bowel syndrome with diarrhea
ACLF: Acute chronic liver failure
AFLD: Alcoholic fatty liver disease
NAFLD: Nonalcoholic fatty liver disease
NASH: Nonalcoholic steatohepatitis
AP: Acute pancreatitis
ZBP1: Z-DNA-binding protein 1
Nt: Nucleotide
Ago2: Argonaute 2
FBXO47: F-box protein 47
EEF1A1: (Eukaryotic translation elongation factor 1 alpha 1)
MDM2: Mouse double minute 2
p53: Tumor protein 53
NELL2: New Epidermal growth factor-like-like 2
MAPK: Mitogen-activated protein kinase
p-ERK: P-JNK, p-p38,extracellular signal-related kinase
TGFB2: Transforming growth factor beta 2
LRP6: Low-density lipoprotein receptor-related protein 6
Wnt: Contraction of 'Wingless'and 'Int'
CACNA1d: Calcium Voltage-Gated Channel Subunit Alpha1 D
GPR78: G protein-coupled receptor 78
PAPSS2: Phosphoadenosine-phosphosulfate synthetase 2
PI3K: Phosphoinositide-3 kinase
AUC: The area under the curve
CEA: Carcinoembryonic antigen
CA199: Carbohydrate antigen 199
CA724: Carbohydrate antigen 724
Dicer1: A type of ribonuclease
EMT: Epithelial-to-mesenchymal transition
LATS2: Large tumor suppressors 2
MALAT1: Metastasis-associated lung adenocarcinoma transcript 1
SRSF2: Serine and arginine rich splicing factor 2
SMC1A: Structural maintenance of chromosomes protein 1
Claudin-1: Senescence-associated Epithelial Membrane Protein 1
NICD1: Notch1 intracellular domain
LGALS1: Galectin 1
MIF: Macrophage migration inhibitory factor
DFS: Disease-free Survival
RPS28: Ribosomal protein mRNAs
NDFIP2: Nedd4 family-interacting protein 2
CEACAM1: Carcinoembryonic antigen-related cell adhesion molecule 1
EIF4A1: Eukaryotic translation initiation factor
TPRG1L: Tumor Protein P63 Regulated 1 Like
NF-κB: C-myc
CDK2: Cell cycle regulators
KIF16: Ruppel-like factor family16
hnRNP: Heterogeneous nuclear ribonucleoproteins
DDX17: HnRNP L-DEAD-box helicase 17
mH2A1: Pre-mRNAstRF-21
UPF1: Up-frameshift proteins
Fn: Fusobacterium nucleatum
GABBR2: GABAB receptor 2
TLR4: Toll-like receptor 4
GABARAP: Gamma-aminobutyric acid receptor-associated protein
TSR1: pre-rRNA processing protein TSR1 homolog
HBV: hepatitis B virus
SIRT1: Sirtuin 1
